# Mental health issues decrease diabetes-specific quality of life independent of glycaemic control and complications: findings from Australia’s living with diabetes cohort study

**DOI:** 10.1186/1477-7525-11-170

**Published:** 2013-10-16

**Authors:** Maria Donald, Jo Dower, Joseph R Coll, Peter Baker, Bryan Mukandi, Suhail AR Doi

**Affiliations:** 1 School of Population Health, University of Queensland, Herston Campus, Brisbane, Queensland, Australia

**Keywords:** Diabetes-specific quality of life, Audit of Diabetes-Dependent Quality of Life (ADDQoL), Type 2 diabetes, Adults

## Abstract

**Background:**

While factors associated with health-related quality of life for people with chronic diseases including diabetes are well researched, far fewer studies have investigated measures of disease-specific quality of life. The purpose of this study is to assess the impact of complications and comorbidities on diabetes-specific quality of life in a large population-based cohort of type 2 diabetic patients.

**Methods:**

The Living with Diabetes Study recruited participants from the National Diabetes Services Scheme in Australia. Data were collected via a mailed self-report questionnaire. Diabetes-specific quality of life was measured using the Audit of Diabetes-Dependent Quality of Life (ADDQoL) questionnaire. The analyses are for 3609 patients with type 2 diabetes. Regression models with adjustment for control variables investigated the association of complications and comorbidities with diabetes-specific quality of life. Next, the most parsimonious model for diabetes-specific quality of life after controlling for important covariates was examined.

**Results:**

The expected associations with better diabetes-specific quality of life were evident, such as increased income, not on insulin, better glycaemic control and older age. However, being single and having been diagnosed with cancer were also associated with better ADDQoL. Additionally, poorer diabetes-specific quality of life was strongly sensitive to the presence of diabetes complications and mental health conditions such as depression, anxiety and schizophrenia. These relationships persisted after adjustment for gender, age, duration of diabetes, treatment regimen, sampling region and other treatment and socio-demographic variables.

**Conclusions:**

A greater appreciation of the complexities of diabetes-specific quality of life can help tailor disease management and self-care messages given to patients. Attention to mental health issues may be as important as focusing on glycaemic control and complications. Therefore clinicians’ ability to identify and mange mental health issues and/or refer patients is critical to improving patients’ diabetes-specific quality of life.

## Background

Diabetes mellitus currently affects about 285 million adults worldwide, with this figure expected to rise to 439 million adults by 2030 [1]. The day-to-day medical management of diabetes for the rapidly increasing number of people diagnosed with the disease is demanding both physically and emotionally and can have an adverse impact on patients’ quality of life [2,3]. Diabetes affects many areas of life, such as diet, employment and leisure. Moreover, the chronic nature of diabetes means that the impacts of the disease may be experienced for many years and its management and treatment can be complex and time consuming. The forecast scale of the epidemic, coupled with the nature of the illness, give good reason to investigate the ways in which diabetes impacts on people’s quality of life.

Researchers have underlined the importance of distinguishing between health-related quality of life, quality of life, and disease-specific quality of life [4,5]. Health-related quality of life measures a patient’s symptoms and functioning, including dimensions such as mobility, pain, or a patient’s ability to care for themself or engage in usual daily activities. Quality of life, on the other hand, is a broad concept encompassing health, but also a person’s values, aspirations, recreational pursuits and interpersonal relationships [6]. Disease-specific quality of life also captures these non-health related aspects of a person’s life but also relates to those aspects directly impacted by the disease. For example in the case of diabetes this includes features such as dietary restrictions, the ability to maintain a working life or to travel despite the inherent complexities of managing diabetes. Disease-specific quality of life measures are generally sensitive to the specific disease’s treatments and complications [7].

A number of disease-specific quality of life measures designed for people with diabetes mellitus have been developed in recent years. Of these, several have been found to have good psychometric properties [7,8], including the Audit of Diabetes-Dependent Quality of Life (ADDQoL) questionnaire [9,10]. Despite recognition of diabetes-specific quality of life as a useful patient reported outcome, as well as numerous studies designed to investigate the psychometric properties of the ADDQoL [11-13], little work has been done to investigate the factors associated with diabetes-specific quality of life in large scale epidemiological studies.

Sundaram and colleagues (2009) have emphasised the need to strengthen the understanding of the impact of various disease and treatment characteristics, and comorbid conditions, on diabetes-specific quality of life, especially as distinguished from health-related quality of life. In one of the few descriptive studies to investigate diabetes-specific quality of life, relying on a small clinic-based convenience sample, it was found that diabetes-specific quality of life was associated with insulin use, glycaemic control, the number of diabetes-related complications and depressive symptomatology, but not obesity [5]. Furthermore, previous research has shown that diabetes-specific quality of life as measured by the ADDQoL distinguishes between insulin treated and non-insulin treated patients, and is sensitive to the presence of diabetes complications [9] but is unaffected by comorbidity unrelated to diabetes [14]. This pattern of associations is consistent with the tenets of disease-specific quality of life measures. However, no study has had a large enough sample size to evaluate individual diabetes complications and comorbidities rather than a simple count, or to test associations using multivariable models.

The purpose of the present study was to assess the diabetes-specific quality of life of a large sample of patients with type 2 diabetes. Specific objectives included: (1) to assess which individual diabetes complications are associated with diabetes-specific quality of life among patients with type 2 diabetes; (2) to assess which, if any, individual comorbidities are associated with diabetes-specific quality of life among patients with type 2 diabetes; and (3) to use a multivariable framework to identify factors independently associated with diabetes-specific quality of life.

## Methods

### Study design

Data reported are from the Living with Diabetes Study, a longitudinal study conducted in the State of Queensland, Australia. Data were collected annually, from 2008 through 2011, via a mailed self-report questionnaire. This paper reports on a cross-sectional analysis of the 2008 baseline data. Details of the methods, baseline characteristics and generalizability of the sample are published elsewhere [15,16].

### Participants

Participants were recruited from the National Diabetes Services Scheme (NDSS), an initiative of the Australian Government administered by Diabetes Australia. It is estimated that the NDSS covers 80% to 90% of the Australian population diagnosed with diabetes [17]. People were eligible to participate in the study if they were aged 18 years or older and had physician-diagnosed type 1 or type 2 diabetes. The study oversampled in three areas of policy interest: an outer metropolitan area, a new suburban development and a coastal agricultural community (all analyses are adjusted for this region-based sampling scheme). A sample of 14439 registrants of the NDSS was invited to participate at baseline in 2008. Completed questionnaires were returned by 3951 participants, yielding a participation rate of 29% after notified deaths and returns to sender were omitted. Ninety-five percent (n=3761) of the Living with Diabetes Study participants had a diagnosis of type 2 diabetes.

Ethics approval for the study was granted by the University of Queensland’s Behavioural and Social Sciences Ethical Review Committee. Written informed consent was obtained from all study participants.

### Measuring diabetes-specific quality of life

The ADDQoL includes two global items; one assesses generic or “present” overall quality of life (measured on a seven point scale: range −3 to +3) and the second assesses diabetes-specific quality of life (measured on a five point scale: range −3 to +1) [9,10]. For both items lower scores reflect poorer quality of life. The ADDQoL also examines the impact of diabetes on 19 specific aspects of life. For five of the domains (work life, holidays, family/relatives, close personal relationship and sex life) respondents may indicate that the domain does not apply to them and the domain score is set to missing. The ADDQoL allows participants to rate the impact of diabetes (positive or negative) on each domain, as well as rate the importance of that domain for their quality of life. The impact score is then multiplied by the importance rating to yield a weighted impact score for each domain (range −9 to +3). An average weighted impact (AWI) score is also calculated for the entire scale by averaging across all applicable domains. In this case, the AWI score was not calculated for 140 respondents due to missing data in 10 or more of the 19 domains.

Several recent reviews of diabetes-specific quality of life instruments conclude that there is good evidence that the ADDQoL is reliable with good face and content validity [7,8,18]. The psychometric properties of the ADDQoL for the Living with Diabetes Study have been published previously and showed that all 19 domains loaded above 0.4 using a forced principal components analysis with a varimax rotation and internal consistency reliability was excellent with a Chronbach’s alpha value of 0.95 [19].

### Complications and comorbidities

The participants were asked to indicate with which of the above, if any, they had ever been diagnosed. Diabetes complications measured were the presence or absence of any of the following: eye disease, kidney disease, nerve damage or neuropathy, heart disease, stroke or transient ischemic attack, erectile dysfunction, poor circulation, foot ulcers, and gangrene or amputation. Participants were also provided with a list of comorbidities not directly related to diabetes (i.e. those that do not share the same pathogenesis or treatment approach as diabetes), including asthma, chronic obstructive pulmonary disease, arthritis, dementia, schizophrenia or psychosis, bipolar disorder/manic-depression, depression, anxiety, substance use disorder, osteoporosis, malignant melanoma, and non-melanoma cancers (lung, prostate, breast, or other).

### Other relevant covariates

After considering the literature on diabetes-specific quality of life, as well as the measures collected in the Living with Diabetes Study, the following variables were examined in addition to the complications and comorbidities in this paper. Patient socio-demographic characteristics included their gender, age, marital status (i.e. never married, married or living with a partner, and secondarily single), and annual household income. Diabetes related covariates included a self-reported measure of glycaemic control (collected by asking patients the result of their most recent HbA1c test), insulin treatment status and the duration of their diabetes. Body mass index (BMI) was measured by asking patients their weight and height. Only 12 study participants reported a BMI in the underweight range and were excluded from these analyses.

### Statistical analysis

The first step in the analysis was to summarise the means and medians to determine the impact of diabetes on each of the 19 ADDQoL domains. The next step was to determine the association between diabetes-specific quality of life as measured by the ADDQoL AWI score and each diabetes complication and comorbid condition separately. Each complication was entered into a regression model that included five control variables, namely gender, age, duration of diabetes, treatment regimen and sampling region. Each comorbidity was also assessed using the same control variables. Results of these partially adjusted models for complications and comorbidities are presented as least squares means in radar plots.

To determine which factors were independently associated with the outcome, a multiple linear regression with a backward-elimination selection procedure was used. Candidates for inclusion in the model were the total number of complications, those comorbidities that were significant in the partially adjusted models as well as other variables thought to be plausibly associated with the outcome. The count of diabetic complications was used rather than each individual complication to reduce the chance of multicollinearity due to associations among the various complications. This is not the case for comorbidities where each illness is not related to the others and an association with diabetes-specific quality of life was not expected. As control variables, sampling region, gender, age, duration of diabetes and treatment regimen were forced into the model regardless of their statistical significance. For all other variables, the criteria for a factor staying in the model was set to p<0.05. Results of the multiple linear regression are presented as least squares means with 95% confidence limits. All analyses were conducted using SAS Version 9.3.

## Results

### Characteristics of respondents

The analyses reported here are for 3609 patients with type 2 diabetes. Forty-four percent (n=1594) of the participants were female. Participants’ age ranged from 22 to 94 years, with an overall mean age of 62.2 years. The mean age of men (63.3 years, 95% CI 62.9-63.8) was significantly greater (p<0.001) than that of women (60.8 years, 95% CI 60.3-61.4). The majority of participants (approximately 62%) were not employed, with 45% reporting that they had retired. While 13.3% of participants had completed university study, 47.5% reported education to year 10 or below. 1.7% of the sample identified themselves as being Aboriginal or Torres Strait Islander Australians.

The mean age at diagnosis of type 2 diabetes was 54.7 years. On average, 7.4 years had elapsed since the date of diabetes diagnosis. Approximately 18% of participants (n=659) required insulin to treat their diabetes (either alone or in combination with oral medications), 61% required glucose-lowering tablets (n=2196) and 21% treated their diabetes with diet and/or exercise alone (n=753).

### Diabetes-specific quality of life

The mean AWI score for the sample was −1.59 (n=3609; SD=1.75; range −9 to 1). The mean for the generic quality of life item was +1.09 (n=3589; SD=0.98; range −3 to 3) and the mean for the generic diabetes quality of life item was −1.07 (n=3587; SD=0.98; range −3 to 1). Overall, all 19 domains were negatively impacted by the presence of diabetes. However examination of the medians showed only 5 domains to be negatively impacted. Freedom to eat as desired was the most impacted aspect of life, and perception regarding the way others react to participants was the least impacted domain (Table 1).

**Table 1:** Impact of type 2 diabetes on each of the 19 domains of the ADDQoL

**Domain**	**Weighted impact score**		
	**N**	**Mean (SD)**	**Median (IQR)**
Freedom to eat	3586	−2.74(2.85)	−2(−4,0)
Sex life	2815	−2.33(2.99)	0(−4,0)
Feelings about future	3529	−2.20(2.86)	−1(−4,0)
Personal relationship	3144	−2.01(2.87)	0(−3,0)
Freedom to drink	3595	−1.83(2.51)	−1(−3,0)
Work life	1560	−1.78(2.49)	0(−3,0)
Holidays	3044	−1.73(2.40)	0(−3,0)
Physical ability	3427	−1.71(2.31)	−1(−3,0)
Family/relatives	3457	−1.68(2.55)	0(−3,0)
Motivation levels	3536	−1.66(2.59)	0(−3,0)
Leisure activities	3421	−1.65(2.22)	−1(−2,0)
Friendships and social life	3508	−1.37(2.25)	0(−2,0)
Financial situation	3552	−1.37(2.38)	0(−2,0)
Travel	3487	−1.33(2.13)	0(−2,0)
Self-confidence	3524	−1.30(2.35)	0(−2,0)
Physical appearance	3509	−1.24(2.27)	0(−2,0)
Dependent on others	3459	−1.13(2.38)	0(−1,0)
Living conditions	3560	−1.07(2.20)	0(−1,0)
People’s reaction to me	3578	−0.57(1.61)	0(0,0)

### The relationship between diabetes complications, comorbidities and diabetes-specific quality of life

**Figure 1:**
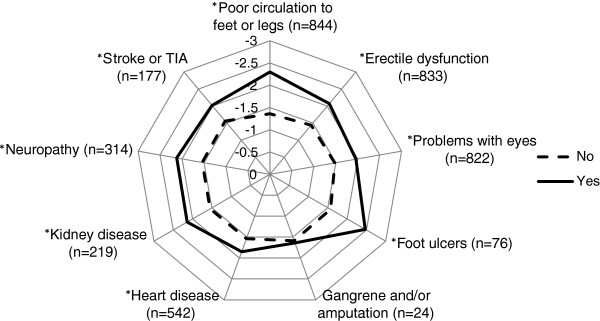
**Diabetes**-**specific quality of life according to the presence or absence of diabetes complications.** Results are expressed as least-square means. Yes = Has ever been told by a doctor or nurse that they have the condition. Analysis included only men for erectile dysfunction, *p <0.05 after adjustment for sampling region, sex, age, duration of diabetes and treatment regimen, n = the number of participants with the condition.

**Figure 2:**
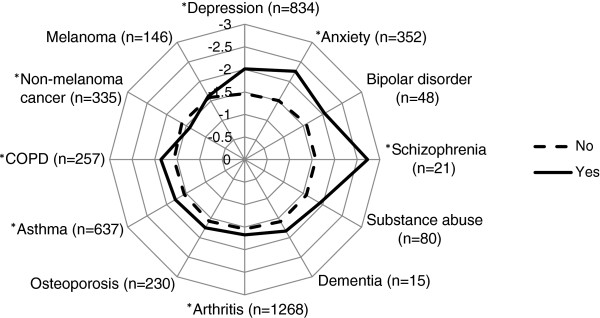
**Diabetes**-**specific quality of life according to the presence or absence of comorbidities.** Results are expressed as least-square means, Yes = Has ever been told by a doctor or nurse that they have the condition, COPD = Chronic Obstructive Pulmonary Disease, *p <0.05 after adjustment for sampling region, sex, age, duration of diabetes and treatment regimen, n = the number of participants with the condition.

Each of the diabetes complications (with the exception of gangrene/amputation) was significantly associated with diabetes-specific quality of life in the partially-adjusted analyses (Figure 1). Although there was a sizable difference in the AWI score between participants with and without a history of gangrene and/or amputation, the difference was not significant, likely due to the small number of participants with this condition. The partially adjusted analyses for the comorbidities showed a history of mental health issues (depression, anxiety, schizophrenia) as well as arthritis, COPD and asthma were associated with poorer diabetes-specific quality of life, while a history of cancer (non-melanoma) was associated with better diabetes-specific quality of life (Figure 2).

### Final multivariable model for diabetes-specific quality of life

The final regression model was significant (F=24.3; P<.0001) and explained 19% (adjusted R^2^) of the variance in diabetes-specific quality of life. A significant gradient was observed for the number of complications and diabetes-specific quality of life. Mental health issues were retained in the model, diabetic patients with a history of such problems reported poorer diabetes-specific quality of life (Table 2). Interestingly, a history of cancer was associated with better diabetes-specific quality of life. Other significant socio-demographic and diabetes related factors associated with better diabetes-specific quality of life included higher income, never having been married, glycaemic control, age and treatment status.

**Table 2:** **Final multivariable regression model for diabetes**-**specific quality of life**

**Variables**	**N (%)**^**a**^	**Adjusted least squares**	**Lower 95% CI**	**Upper 95% CI**	**p**-**values**
		**Mean**			
**Complications and comorbidites**					
Number of complications^b^					<0.001
0	1579 (46.1)	−1.25	−1.33	−1.17	
1	828 (24.2)	−1.56	−1.66	−1.45	
2	518 (15.1)	−1.92	−2.06	−1.78	
3	284 (8.3)	−2.17	−2.36	−1.98	
4 or more	215 (6.3)	−2.50	−2.72	−2.27	
Depression					0.001
No	2615 (76.4)	−1.53	−1.59	−1.47	
Yes	809 (23.6)	−1.75	−1.87	−1.64	
Anxiety					<0.001
No	3081 (90.0)	−1.54	−1.59	−1.48	
Yes	343 (10.0)	−1.97	−2.15	−1.79	
Schizophrenia					0.016
No	3405 (99.5)	−1.58	−1.63	−1.52	
Yes	19 (0.5)	−2.47	−3.20	−1.75	
Non-melanoma cancers					0.008
No	3107 (90.7)	−1.60	−1.66	−1.55	
Yes	317 (9.3)	−1.35	−1.53	−1.18	
**Other significant demographic and clinical covariates**					
Household income					<0.001
<$20 K	925 (27.2)	−1.82	−1.93	−1.71	
20 K – 40 K	863 (25.2)	−1.50	−1.61	−1.39	
40 K – 80 K	772 (22.6)	−1.42	−1.53	−1.30	
80 K – 120 K	309 (9.0)	−1.36	−1.54	−1.17	
120 K – 160 K	108 (3.2)	−1.25	−1.55	−0.95	
>160 K	67 (2.0)	−0.91	−1.30	−0.53	
Missing/do not know	380 (11.1)	−1.92	−2.08	−1.76	
Marital status					0.018
Never married	208 (6.1)	−1.32	−1.54	−1.09	
Co-habiting	2450 (71.6)	−1.62	−1.69	−1.56	
Secondarily single	766 (22.4)	−1.52	−1.64	−1.40	
HbA1c result					<0.001
Under 6.5%	796 (23.3)	−1.38	−1.49	−1.27	
6.5% - 7.0%	891 (26.0)	−1.57	−1.67	−1.46	
7.1% - 8.0%	746 (21.8)	−1.62	−1.73	−1.50	
Over 8.0%	453 (13.2)	−1.95	−2.10	−1.80	
Do not know	538 (15.7)	−1.55	−1.68	−1.41	
Age quartile					<0.001
Q1: 22–55 y	858 (25.1)	−2.07	−2.18	−1.95	
Q2: 56–62 y	871 (25.4)	−1.67	−1.78	−1.56	
Q3: 63–69 y	877 (25.6)	−1.43	−1.53	−1.32	
Q4: 70–94 y	818 (23.9)	−1.15	−1.27	−1.03	
Treatment status					<0.001
Insulin requiring	630 (18.4)	−2.01	−2.15	−1.88	
Oral medications	2078 (60.7)	−1.58	−1.64	−1.51	
Diet and/or exercise only	716 (20.9)	−1.22	−1.34	−1.10	

Model is adjusted for the five control variables: Age and treatment status were significant; sampling region, sex and duration of diabetes were non-significant and are not shown.

^a^185 participants (5.1%) had missing data for one or more covariates and are not included in this analysis.

^b^includes eye disease, kidney disease, nerve damage or neuropathy, heart disease, stroke or transient ischemic attack, erectile dysfunction, poor circulation, foot ulcers, and gangrene or amputation.

## Discussion

Diabetes has the greatest overall negative impact on patients’ freedom to eat as they wish, and the least negative impact on their perception concerning the way others in society react to them. The negative impact that the loss of dietary flexibility has on patients’ diabetes-specific quality of life is of particular interest given the ubiquitous nature of this finding across different contexts and research studies [10-13]. It likely refers not only to dietary restrictions around healthful eating and weight loss but also the need to regularly monitor the relationship between food intake, energy expenditure and blood glucose levels. This poses something of a challenge to the way the management of type 2 diabetes is undertaken because while dietary change may help delay outcomes associated with poorer quality of life such as disease progression and the onset of diabetes complications [20,21], it is itself associated with decreased quality of life. One method available to health care providers to encourage good self-care among diabetic patients is to emphasise the increased potential for developing diabetes complications with poor diet and poor glycaemic control. However, undue emphasis on this association may be counterproductive and could result in a negative impact on self-care and consequently glycaemic control [22].

Indeed, our study has confirmed that diabetes complications are associated with poorer diabetes-specific quality of life. Each individual diabetes complication raised the likelihood of patients reporting a lower diabetes-specific quality of life. Moreover, each additional complication further reduced diabetes-specific quality of life even after controlling for other important covariates. Unlike complications however, the presence of comorbidities does not influence diabetes-specific quality of life after controlling for relevant covariates. The clear exceptions were mental health issues and cancer. The former confirms previous reports [23].

Perhaps these mental health problems impact on disease management, and in particular a patient’s capacity for self-care more so than the physically orientated comorbidities. There is evidence to suggest that depression is associated with a decrease in some self-care behaviours [24,25]. Alternatively, symptoms of anxiety and depression adversely affect the degree of acceptance of illness and significantly lower the quality of life of those with diabetes [26]. There is accumulating evidence to suggest that diabetes, depression and quality of life are closely interrelated and that diabetes is causally related to depression and vice versa [27]. Whether depression and anxiety should be considered complications of diabetes rather than comorbidities is of interest. In either case, it is clear that additional attention must be paid to diabetic patients with mental health issues such as anxiety and depression in order to ensure that they enjoy a quality of life comparable to that of those without these mental health problems. Improvements to both quality of life and disease management could be achieved by improving the identification and management of mental health problems among people with diabetes [2,23].

Also of interest in relation to comorbidities was the finding that diabetes-specific quality of life was significantly better in cancer survivors than the rest of the diabetic population. Previous research has shown that individuals with diabetes and cancer have a significantly lower health-related quality of life than those with either condition alone [28]. The findings reported here are not incompatible with this but instead reinforce the importance of distinguishing between health-related quality of life and disease-specific quality of life. The finding most likely reflects the fact that our diabetic cancer survivors rated their diabetes-specific quality of life as better than their non-cancer peers because cancer-specific decreases in health status are not attributed to diabetes and/or the adverse impact of cancer-specific characteristics on quality of life outweigh those related to diabetes.

The finding that better metabolic control was associated with better diabetes-specific quality of life independent of complications suggests that efforts to achieve optimal metabolic control are justified on quality of life grounds as well as clinical grounds. This however needs to be reconciled with the seemingly conflicting finding that dietary restrictions, an important aspect of maintaining optimal metabolic control, has such an adverse impact on diabetes-specific quality of life. Rubin and Peyrot (1999) point out that it seems reasonable to conclude that the benefits of good glycaemic control offset the constraints or burden imposed by the more demanding self-care regimen required to maintain it especially in the longer-term. Alternatively, “current” glycaemic control may simply serve as a marker of mental health and thus the ability to maintain “current” good control [25,29]. This should not be confused with the “duration of good control” that would be expected to correlate with better health outcomes, including quality of life. It seems reasonable to conclude that while mental health issues (such as depression) may interfere with effective diabetes self-management, and lead to deterioration in glycaemic control [30], at the same time, good control of diabetes over the longer-term cumulatively leads to better quality of life measures [3]. Future studies need to take into account this distinction between current glycaemic status and prior glycaemic control.

The direction of the age effect was unexpectedly in favour of older age such that it was associated with better diabetes-specific quality of life. Satisfaction with family life, vocational and financial situations improve with age [31] and perhaps expectations decline with age too. This lowering of expectations may be an important mechanism by which older adults maintain satisfaction with their lives or certain aspects of their lives despite declining health. Alternatively, as there is generally an increased prevalence of health conditions amongst older persons, society may provide greater support for adjusting to living with diabetes for older people than it does for younger people with diabetes, whose social networks may provide fewer opportunities for social support.

This study has both strengths and limitations. There are few studies designed to explore how diabetes impacts on the lives of people living with type 2 diabetes with a sample size of the magnitude and representativeness of the Living with Diabetes Study. For example, we have found no previous study with adequate statistical power to confirm the association between individual diabetes complications and diabetes-specific quality of life after controlling for important covariates. Findings are however limited by the fact that complications and comorbid diagnoses were self-reported and therefore reliant on recall. The presence of some comorbidities were likely (e.g. mental health disorders, substance abuse) or almost certainly (e.g. dementia) to be underreported by the patients. Furthermore, patients were asked whether a doctor or nurse had ever told them that they had any of a predefined list of comorbid conditions. While these conditions were for the most part chronic in nature, it is possible that in some cases the condition may not have had any current bearing on the patient, for example having suffered from asthma as a child, or having been successfully treated for cancer many years ago. Also, an overlap between quality of life measures and measures of depressive symptomatology has been noted [32]. Yet in the current study this effect will be attenuated by our use of a diabetes-specific measure of quality of life that has less commonality of items that measure depression than for example a health-related quality of life measure.

Finally, the response rate for participants consenting to participate in the research was low, yet consistent with research showing that participation rates in large cohort studies appear to be declining [33]. In a detailed analysis of respondents versus non-respondents, it was shown that individuals were less likely to participate in the Living with Diabetes Study if they were younger or older than those aged 50 to 69 years or had identified themselves as being Aboriginal or Torres Strait Islander Australians, whereas there was no difference in relation to gender, length of time since diagnosis or socio-economic status [16]. David and colleagues (2011) observed that when disease registers are used to recruit patients, the generalizability of a study’s findings to the target population is very much dependent on register coverage and the quality of its database. Given the coverage of the NDSS is estimated to be between 80% and 90%, which is higher than most diabetes registers, it has the potential to produce sampling frames of a higher data quality than most.

## Conclusions

The findings from this large cohort study about diabetes-specific quality of life have important implications for the care of those with type 2 diabetes. In particular, complications and poor glycaemic control independently serve to decrease diabetes-specific quality of life. While dietary control and the prevention of complications, for example, are important aspects of the management of the condition, loss of the freedom to eat as desired was the most negatively affected aspect of quality of life among the study participants. This creates something of a dilemma for health care providers in that they need to educate patients about the longer-term link between healthful eating, better glycaemic control and reduced risk of diabetes complications yet an over-emphasis on the need for healthful eating may impede quality of life. Mental health issues also need to be addressed as they were an important contributor to decreased diabetes-specific quality of life. Finally, comorbidities seem to play a much smaller role and indeed cancer survivors may even paradoxically have better diabetes-specific quality of life. Since a focus on glycaemic control and complications is already a routine aspect of diabetes care, improvement in mental health care can go a long way towards increasing diabetes-specific quality of life in these populations.

## Abbreviations

ADDQoL: Audit of diabetes-dependent quality of life; NDSS: National diabetes services scheme; AWI: Average weighted impact score; HbA1c: Glycated hemoglobin; BMI: Body mass index.

## Competing interests

The authors declare they have no competing interests.

## Authors’ contributions

MD conceived of the study and drafted the manuscript. JD, BM and SD participated in the study’s design and coordination and helped to draft the manuscript. JC and PB analysed the data. All authors critically reviewed the manuscript and approved the final manuscript.
